# Services for perinatal patients with opioid use disorder: a comprehensive Baltimore City-wide 2023 assessment

**DOI:** 10.1186/s13722-024-00507-0

**Published:** 2024-10-15

**Authors:** Jessica A. Ratner, Jennifer H. Kirschner, Brittney Spencer, Mishka Terplan

**Affiliations:** 1 Division of Addiction Medicine, Johns Hopkins School of Medicine, Baltimore, MD USA; 2 Baltimore City Health Department, Baltimore, MD USA; 3 Friends Research Institute, Baltimore, MD USA

**Keywords:** Health services, Public health, Maternal mortality, Overdose, Opioid use disorder, Pregnancy

## Abstract

**Background:**

Overdose is a leading cause of maternal mortality; in response, maternal mortality review committees have recommended expanding substance use disorder (SUD) screening, improving collaboration between obstetric and SUD treatment providers, and reducing fragmentation in systems of care. We undertook an analysis of the perinatal SUD treatment landscape in Baltimore, Maryland in order to identify barriers to treatment engagement during pregnancy and the postpartum period and guide system improvement efforts.

**Methods:**

We conducted a survey of seven birthing hospitals, 31 prenatal care practices, and 108 SUD treatment providers in Baltimore from April-June 2023. Organizations were asked to quantify care for perinatal patients with opioid use disorder (OUD) as well as about screening, service availability, referral practices, and support needed to improve care.

**Results:**

61% of the 145 contacted organizations responded. Birthing hospitals reported caring for pregnant persons with OUD with greater frequency than prenatal care practices or SUD treatment programs. Most birthing hospitals and prenatal care practices reported screening for OUD at intake, but the minority reported using validated tools. Service availability varied by type of organization and type of service. In general, prenatal care practices offered the fewest number of SUD-related services. Most SUD treatment programs that offered buprenorphine or methadone to the general population also offered these medications to pregnant patients. Withdrawal management for comorbid alcohol/benzodiazepine use disorders during pregnancy was more limited. The majority of birthing hospitals and prenatal care practices reported offering neither direct naloxone distribution nor prescriptions. Few SUD treatment programs offered tailored services for perinatal patients or for parents of young children, and many programs do not permit children onsite. Respondents reported high levels of interest in education and consultative support on SUD treatment in pregnancy within obstetric settings and on pregnancy-related medical concerns within SUD programs.

**Conclusions:**

This project provides a comprehensive picture of services available for treatment of perinatal OUD in a major US city. Results have served as a guide for ongoing citywide system improvement efforts by our project team and offer a model for other jurisdictions hoping to strengthen services for perinatal OUD and reduce maternal mortality.

**Supplementary Information:**

The online version contains supplementary material available at 10.1186/s13722-024-00507-0.

## Background

Overdose is a leading cause of maternal mortality in the United States [1–4] and the most common cause of maternal death in the state of Maryland [5] and the city of Baltimore (unpublished data). Opioid use disorder (OUD) is common in pregnancy and postpartum; almost 3% of pregnant and postpartum Medicaid enrollees in the US have documented OUD [6]. Nationwide, death due to overdose during pregnancy or within the first year postpartum has nearly doubled during recent years, driven by rising overdoses related to synthetic opioids and stimulants [7–9]. The postpartum period, particularly 6 months or more after delivery, is the highest risk time for fatal overdose [8, 10].

Standard of care for OUD in pregnancy includes medications (i.e., buprenorphine or methadone) [11–13]. Medication for OUD (MOUD) is associated with increased retention in substance use disorder (SUD) treatment and reductions in overdose as well as increased prenatal care utilization, lower rates of preterm birth and low birth weight, and maintenance of custody [10, 14–17]. Longer duration of treatment with MOUD during pregnancy increases postpartum treatment retention [14, 17–19].

However, roughly half of pregnant patients with OUD do not receive MOUD, and fewer than two thirds of those who receive treatment in pregnancy remain in treatment postpartum [20–23]. Many barriers limit access to and engagement in OUD treatment for this population, including lack of service availability during pregnancy, siloing of obstetric and SUD services, fear of legal consequences/custody loss, comorbid mental health conditions, stigma, and feelings of guilt/shame [13, 24–29]. Treatment discontinuation and overdose risk may be particularly high postpartum due to added factors of loss of insurance coverage, competing demands of caring for a new family, and, in some cases, custody loss [29–31].

Through review of fatal maternal overdose cases, maternal mortality review committees (MMRCs) offer insight into potential avenues to improve outcomes for this population. Common themes among MMRC recommendations both nationwide and locally in Maryland and Baltimore have included: promotion of standardized screening for SUD during pregnancy; increasing comfort among obstetric providers in caring for patients with SUD and among SUD providers in caring for patients during pregnancy; improving navigation supports and reducing fragmentation within systems of care; anti-stigma training for providers and the broader community; and adoption of family-friendly policies/practices across organizations [5, 31, 32].

Public health departments and other governmental and quasi-governmental agencies can play a vital role in addressing barriers to treatment and reducing overdose mortality among perinatal patients through policies and programming that focus on these MMRC priority areas. Motivated by the rate of maternal overdose deaths in Baltimore as well as local MMRC recommendations, our project team, a public health-academic partnership, aimed to assess availability of substance use services for pregnant and postpartum people with OUD across multiple settings within the local health care system. The project’s ultimate goal was to identify system strengths and gaps, as well as avenues for quality improvement and collaboration to reduce maternal overdose mortality.

## Methods

To ensure comprehensive assessment of services available to pregnant and postpartum patients with OUD in Baltimore, the project team developed a systematic outreach list that included a wide range of healthcare organizations with potential contact with this population. Organizational categories were defined as: (1) birthing hospitals, (2) prenatal care practices, and (3) SUD treatment providers. The SUD treatment provider category was divided into the following sub-categories: (a) opioid treatment programs (OTPs), (b) office-based buprenorphine providers (OBOT), (c) residential programs, (d) state-certified recovery residences, (e) withdrawal management or stabilization programs, and (f) syringe services programs (SSPs). Individual institutions, organizations, and programs (hereafter, referred to as “organizations”) were identified using lists of certified programs maintained by city and state health departments and the local behavioral health authority. For categories for which no list was available (i.e., OBOT, withdrawal management programs), the project team generated a list of known organizations based on their local experience. Several programs known to the project team as providing services to this population but missing from maintained lists were added to the outreach list.

Using either listed or publicly available contact information, project team members reached out to each organization to identify an appropriate respondent and confirm their contact information. Organizations (except birthing hospitals) were asked to identify a medical or administrative leader who could answer questions about service availability, common referrals, and barriers to care for pregnant individuals with SUD. Birthing hospitals were asked to identify the lead social worker in their obstetrics unit. Organizations no longer in operation, incorrectly included on initial lists (e.g., reporting no prenatal care offered), or identified as serving men-only during the initial contact confirmation process were removed from the outreach list. Programs for whom no individual respondent could be identified were also removed.

**Fig. 1:**
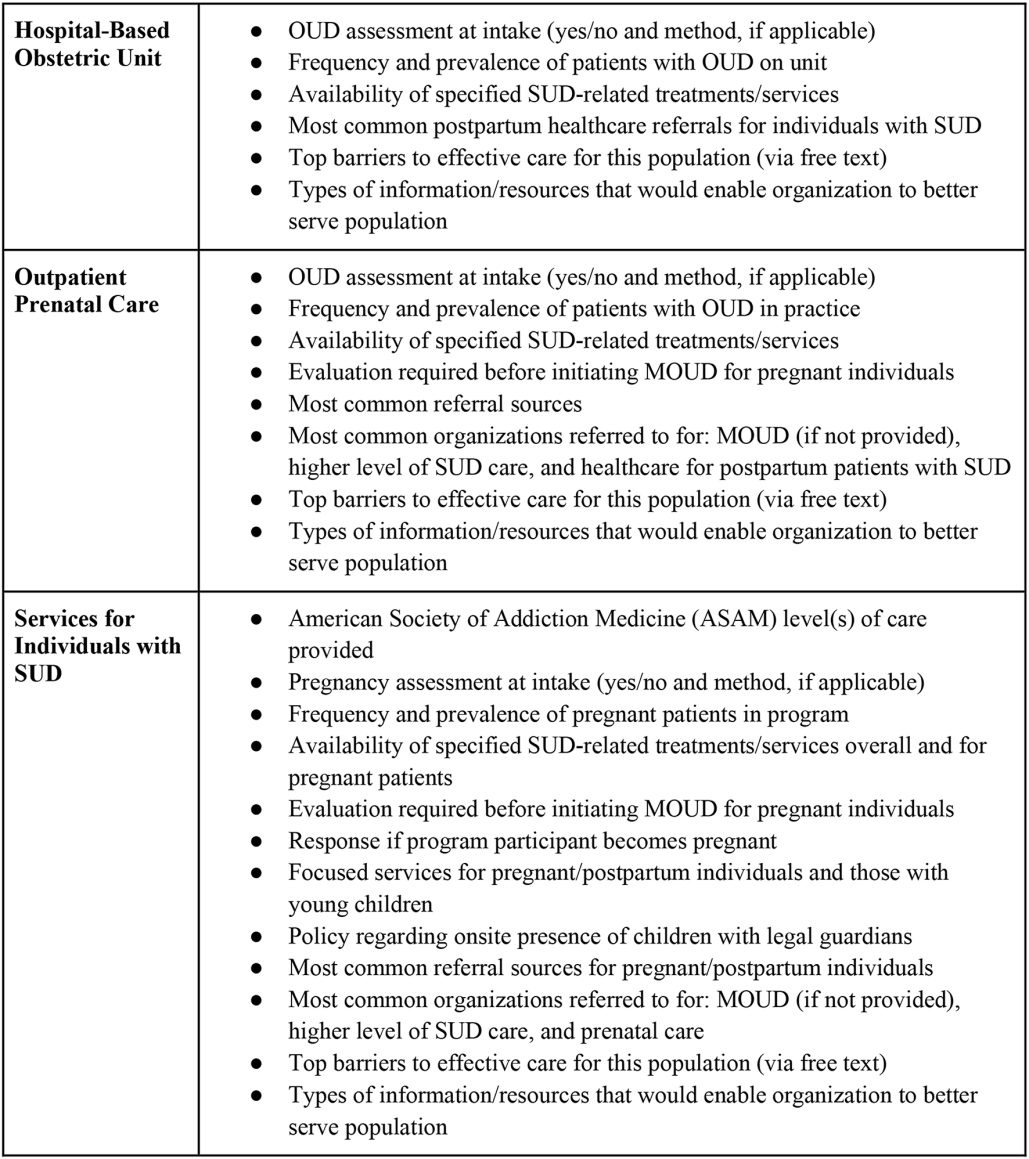
Survey Question Topics by Organization’s Self-Identified “Primary Mission(s)”

A survey instrument was developed to assess each organization’s substance use-related service availability, common referrals and partners, and barriers/needs to care effectively for pregnant and postpartum patients with SUD. Survey questions were specifically drafted to address the public health crisis of maternal deaths by assessing MOUD, naloxone, and linkage to care. Questions were piloted with healthcare and public health professionals with obstetrics and SUD expertise representing 3 different healthcare institutions. The final survey was programmed into and distributed via REDCap (Research Electronic Data Capture), a secure, web-based software platform designed to support data capture for research and quality improvement [33]. Specific questions asked of each organization were determined by a skip pattern based on the organization’s self-identified “primary mission(s)” (“services for individuals with substance use disorder,” “outpatient prenatal care,” and/or “hospital-based obstetrics”) in order to maximize question relevance. See Fig. 1 for a detailed list of topics covered by survey questions and the Supplement for a copy of the complete survey instrument.

The project team implemented a systematic strategy to ensure broad reach and maximize response rates. Initial outreach to all organizations was conducted by email. Respondents identified during the contact confirmation phase were asked to complete the survey or forward to a more appropriate respondent. The survey instrument was preceded by language informing respondents that the survey was voluntary, would take ten minutes to complete, and would not collect personal health information. Participants were given the option to confidentially provide their name and work contact information for possible follow-up. Respondents were not compensated for participation. Up to four additional attempts at contact were made over a two-month period by either email or phone before marking non-response. All data was collected from April-June 2023.

For analysis, descriptive statistics (i.e., frequencies) were calculated using REDCap reports and Excel. Several organizations were included in more than one category–for example, a SUD treatment program on both the residential and certified-recovery residency lists. Responses for these organizations were included only once in analyses for main organizational categories (e.g., SUD programs) but were included in analyses for each of the applicable sub-categories (e.g., residential and recovery residences). Missing values were limited as the survey primarily utilized forced-choice questions including a “don’t know/unsure” answer option. The Johns Hopkins School of Medicine institutional review board deemed this project to be quality/systems improvement work and, thus, exempt from review.

## Results

Among the 145 organizations that were contacted, 89 responded, comprising 61% of the total sample. Response rates varied by organization type, with responses from 100% of the seven contacted birthing hospitals (*n* = 7), 65% of the 31 contacted prenatal care practices (*n* = 20), and 58% of the 108 contacted substance use-related services programs (*n* = 63; sum of three categories exceeds total N due to one program listed in both prenatal care and SUD categories). Among SUD programs, response rates ranged from 24% of recovery residences to 100% of withdrawal management/stabilization units.

**Table 1:** Frequency of caring for pregnant patients with OUD

	Always Row % (*n*)	Frequently Row % (*n*)	Sometimes Row % (*n*)	Rarely Row % (*n*)	Never Row % (*n*)	Unsure Row % (*n*)
Birthing Hospitals (*n* = 7)	28.2% (2)	42.9% (3)	28.2% (2)	0	0	0
Prenatal Care Practices (*n* = 20)	20.0% (4)	15.0% (3)	30.0% (6)	35.0% (7)	0	0
SUD Programs (*n* = 63)*	6.3% (4)	12.7% (8)	33.3% (21)	34.9% (22)	7.9% (5)	4.8% (3)
OBOT (*n* = 12)	0	25.0% (3)	16.7% (2)	50.0% (6)	8.3% (1)	0
OTP (*n* = 21)	4.8% (1)	19.0% (4)	52.4% (11)	23.8% (5)	0	0
SSPs (*n* = 7)	0	0	28.6% (2)	28.6% (2)	14.3% (1)	28.6% (2)
Residential (*n* = 12)	0	8.3% (1)	25.0% (3)	50.0% (6)	8.3% (1)	8.3% (1)
Recovery Residence (*n* = 8)	37.5% (3)	0	25.0% (2)	12.5% (1)	25.0% (2)	0
Withdrawal Management(*n* = 6)	0	16.7% (1)	33.3% (2)	50.0% (3)	0	0

*Total N for SUD programs is less than sum of subcategories due to several programs falling into multiple categories

Organizations were asked to indicate the frequency with which they provided care for pregnant patients with OUD on a five-point Likert scale, ranging from “always” to “never” (Table 1), as well as the average number of pregnant patients with OUD treated per month. Birthing hospitals reported caring for this population with greater frequency (71.4% always or frequently) than prenatal care practices (35.0% always/frequently) or SUD programs (19.0% always/frequently). Among SUD programs, OTPs and recovery residences reported more frequently caring for this population, with only 23.8% of OTPs and 37.5% of recovery residences selecting “rarely” or “never.” Most birthing hospitals reported treating 10–20 pregnant patients per month with OUD, whereas the majority of PNC practices and SUD programs reported treating five or fewer.

**Table 2:** OUD and pregnancy screening practices

	Birthing Hospitals (*n* = 7) Column % (*n*)	Prenatal Care Practices (*n* = 20) Column % (*n*)	SUD Programs (*n* = 63) Column % (*n*)
**Assess for OUD at intake**	100% (7)	95.0% (19)	N/A
With informal verbal/written screening	57.1% (4)	65.0% (13)	N/A
With validated verbal/written tool	28.6% (2)	45.0% (9)	N/A
With urine toxicology	85.7% (6)	60.0% (12)	N/A
With urine toxicology only	14.3% (1)	0	N/A
**Assess for pregnancy at intake**	N/A	N/A	85.7% (54)
With urine pregnancy test	N/A	N/A	60.3% (38)

Table 2 shows reported screening practices. Birthing hospitals and PNC practices were asked whether and how they screen for OUD at intake for services. Almost all responding organizations reported screening for OUD; most reported using urine toxicology (85.7% of birthing hospitals, 60.0% of PNC practices) and informal verbal/written screening (57.1% of birthing hospitals, 65.0% of PNC practices). One birthing hospital reported using only urine toxicology to screen for OUD. SUD programs were asked about whether and how they screen for pregnancy at intake. While 85.7% of programs assessed pregnancy status, only 60.3% utilized urine pregnancy tests. Urine pregnancy testing was most frequently reported by OTPs (100%), withdrawal management programs (83.3%), and residential programs (80.0%).

**Table 3:** SUD-Related service availability during pregnancy

	Birthing hospitals (*n* = 7)	Prenatal care practices (*n* = 20)	SUD programs (*n* = 63)*		
	Available column % (*n*)	Available column % (*n*)	Available column % (*n*)	Unavailable to pregnant patients only column % (*n*)	Unavailable to all patients column % (*n*)
Buprenorphine initiation	57.1% (4)	25.0% (5)	66.7% (42)	6.3% (4)	22.2% (14)
Buprenorphine maintenance	100% (7)	35.0% (7)	69.8% (44)	6.3% (4)	22.2% (14)
Methadone initiation	57.1% (4)	0	47.6% (30)	3.2% (2)	47.6% (30)
Methadone maintenance	100% (7)	0	55.6% (35)	3.2% (2)	39.7% (25)
Naltrexone initiation	42.9% (3)	10.0% (2)	34.9% (22)	11.1% (7)	50.8% (32)
Naltrexone maintenance	57.1% (4)	10.0% (2)	36.5% (23)	9.5% (6)	49.2% (31)
Withdrawal management – alcohol/benzos	71.4% (5)	10.0% (2)	34.9% (22)	4.8% (3)	57.1% (36)
Withdrawal management – opioids	71.4% (5)	0	38.1% (24)	7.9% (5)	49.2% (31)
Brief intervention (SBIRT)	100% (7)	50.0% (10)	68.9% (44)	1.6% (1)	25.4% (16)
Peer recovery support	100% (7)	25.0% (5)	74.6% (47)	1.6% (1)	23.8% (15)
Non-peer treatment linkage services	71.4% (5)	40.0% (8)	91.9% (57)	0	3.2% (2)
Individual SUD counseling	0	25.0% (5)	85.2% (52)	1.6% (1)	14.3% (9)
Group SUD counseling	0	15.0% (3)	74.6% (47)	1.6% (1)	22.2% (14)
Naloxone – direct distribution	14.3% (1)	10.0% (2)	69.8% (44)	3.2% (2)	42.9% (27)
Naloxone – providing prescriptions	42.9% (3)	35.0% (7)	50.8% (32)	3.2% (2)	42.9% (27)
Naloxone – prescribe OR distribute	42.9% (3)	35.0% (7)	84.1% (53)	3.2% (2)	12.7% (8)
Other harm reduction services (e.g., providing sterile supplies)	14.3% (1)	5.0% (1)	34.9% (22)	1.6% (1)	58.7% (37)

*SUD program columns do not always add up to 100%, as programs could mark “Unsure/Don’t Know”

Birthing hospitals and PNC practices were asked to indicate the availability of specific SUD-related services; SUD programs were asked to specify whether these services were available to all patients, pregnant patients only, non-pregnant patients only, or unavailable to all patients (Table 3). Regarding medications for opioid use disorder (MOUD), all birthing hospitals reported capacity to continue buprenorphine and methadone for patients taking these medications at the time of admission, but fewer (57%) reported that MOUD initiation was available. 35% of PNC practices reported offering buprenorphine maintenance, while only 25% offered buprenorphine initiation.

Most SUD programs that offered buprenorphine or methadone reported availability to all patients regardless of pregnancy status, with some notable exceptions. In particular, 76% of responding OTPs reported offering buprenorphine, and none of these programs limited this service based on pregnancy status. One of the 21 responding OTPs reported that their program did not offer methadone during pregnancy. In addition, of the five withdrawal management programs that offered buprenorphine initiation/maintenance to the general population, one of these programs denied initiation and one denied both initiation and maintenance to pregnant people. Four withdrawal management programs offered methadone maintenance and two offered methadone initiation overall; none of these programs limited methadone based on pregnancy status. SUD programs were asked to indicate whether they required obstetrics evaluation prior to initiating MOUD–60.4% reported requiring no obstetric evaluation, 4.2% required ultrasound, 18.8% required outpatient obstetric evaluation, 2.1% required inpatient hospitalization, and 18.8% were unsure.

Withdrawal management services were less widely available than MOUD, with more organizations offering opioid than alcohol/benzodiazepine withdrawal management. Five of seven birthing hospitals reported offering management of opioid and alcohol/benzodiazepine withdrawal. Only 10% of PNC practices offered opioid withdrawal management, and none offered alcohol/benzodiazepine withdrawal management. Among SUD programs, two of five withdrawal management programs offering alcohol/benzodiazepine withdrawal management reported restricting this service to non-pregnant patients only.

Linkage to treatment via SBIRT (i.e., screening, behavioral intervention and referral to treatment), peer recovery support, and/or non-peer treatment linkage services was most widely available at birthing hospitals and SUD programs. Among PNC practices, only half conducted SBIRT, 25% offered peer recovery support, and 40% offered non-peer treatment linkage.

Harm reduction services, including naloxone provision, were less often available at birthing hospitals and prenatal care practices than at SUD programs. Nearly two-thirds of birthing hospitals and PNC practices reported neither prescribing nor distributing naloxone, whereas only 16% of SUD programs reported neither service. Other harm reduction services (e.g., distribution of sterile supplies) were reported by 35% of SUD programs but only 14% of birthing hospitals and 5% of PNC practices.

**Table 4:** Availability of family-focused practices and policies within SUD Programs

	Services for perinatal patients^ Row % (*n*)	Services for parents^#^ Row % (*n*)	Housing available during pregnancy Row % (*n*)	Children allowed onsite w/ legal guardian Row % (*n*)
SUD Programs Overall (*n* = 63)*	19% (12)	14.3% (9)	44.4% (28)	44.4% (28)
OBOT (*n* = 12)	16.7% (5)	8.3% (1)	8.3% (1)	50.0% (6)
OTP (*n* = 21)	23.8% (5)	9.5% (2)	23.8% (5)	57.1% (12)
SSPs (*n* = 7)	14.3% (1)	14.3% (1)	28.6% (2)	57.1% (4)
Residential (*n* = 12)	16.7% (2)	16.7% (2)	91.7% (11)	16.7% (2)
Recovery Residence (*n* = 8)	25.0% (2)	25.0% (2)	87.5% (7)	50.0% (4)
Withdrawal Management (*n* = 6)	0	16.7% (1)	66.7% (4)	0

* Total N for SUD programs is less than sum of subcategories due to several programs falling into multiple categories

^ Defined as “pregnant or within 1 year of birth, termination, or pregnancy loss”

# Defined as “individuals with children under age 5”

To assess specific focus on the perinatal population as well as family-friendliness, SUD programs were asked several questions about tailored services (Table 4). The minority of programs reported having specialized services for perinatal patients or for parents. Among the programs offering inpatient/residential level services, 92% of residential programs, 88% of recovery residences, and 67% of withdrawal management units reported offering housing to pregnant patients. Children were allowed on-site more often among outpatient programs (50% OBOT, 57% OTP, 57% SSPs) than inpatient programs (none of the withdrawal management programs, 17% residential, 50% recovery residences).

**Table 5:** Information/Resources Needed by Organizations to Better Serve Perinatal Patients with OUD

	Birthing Hospitals (*n* = 7) Column % (*n*)	Prenatal Care Practices (*n* = 20) Column % (*n*)	SUD Programs (*n* = 63) Column % (*n*)
Education on meds for OUD in pregnancy	57.1% (5)	30.0% (6)	36.7% (22)
Education on other SUD treatment in pregnancy	85.7% (6)	35.0% (7)	43.3% (26)
Education on pregnancy-related medical concerns	85.7% (6)	25.0% (5)	46.7% (28)
Specialist consultation for SUD treatment in pregnancy	85.7% (6)	40.0% (8)	38.3% (23)
Specialist consultation for pregnancy-related medical concerns	57.1% (4)	5.0% (1)	43.3% (26)
Increased availability of specific resources for patients	85.7% (6)	30.0% (6)	41.7% (25)
Support for implementation for clinical workflows or policies	42.9% (3)	5.0% (1)	13.3% (8)
Onsite sexual health services provided by external partner	28.6% (2)	5.0% (1)	23.3% (14)
Onsite SUD treatment services provided by external partner	57.1% (4)	15.0% (3)	13.8% (8)
Ability to distribute naloxone to individuals directly	14.3% (1)	0	16.7% (10)

Respondents were asked “What information or resources would enable your organization to better serve this population?” and could select among answer options that included resources potentially available through the city health department or community partners (Table 5). Availability of resources for patients was among the top four responses for all three organization categories (86% birthing hospitals, 30% PNC practices, 42% SUD programs). Education/specialist consultation related to MOUD and other SUD treatment were among the most commonly endorsed by birthing hospitals and PNC practices. Conversely, education/specialist consultation on pregnancy-related medical concerns was among the most commonly endorsed by SUD treatment programs.

## Discussion

This project provides a broad look at SUD-related services available to pregnant and postpartum patients across both obstetric and SUD treatment settings in Baltimore, Maryland. To our knowledge, it is the first comprehensive, cross-disciplinary assessment of perinatal OUD service availability in a major US city.

Overall, our results show that most birthing hospitals in Baltimore often care for perinatal patients with OUD, but the majority of PNC practices and SUD programs in the city care for this population less frequently. This imbalance may be driven by several factors including the greater number of PNC and SUD programs available to patients relative to birthing hospitals, preferential referral to specialty outpatient programs, lack of patient engagement in care prior to delivery, and/or underdiagnosis/lack of identification of SUD in the outpatient setting. Additionally, our data show considerable variability in screening practices and in service availability (both by type of organization and by type of service). Birthing hospitals and SUD programs generally provide more SUD-related services than PNC practices. MOUD and linkage to treatment are generally more available than withdrawal management and harm reduction services.

Our data demonstrate a lack of consistent validated SUD screening, availability of MOUD, and naloxone accessibility in prenatal/obstetric settings, representing significant missed opportunities for treatment engagement and overdose prevention. While most birthing hospitals and PNC practices reported screening in some way for OUD, organizations frequently utilized informal screening and/or urine toxicology. Notably, our results show minimal improvement in the decade since prior statewide assessment demonstrated that a minority of obstetric settings used validated substance use screening tools, considered standard of care by many professional and governmental organizations [11–13, 34]. Informal approaches may lack reliability and introduce potential for bias, and urine toxicology may be used punitively [35–37]. Additionally, while all birthing hospitals reported the ability to continue MOUD for patients, two of seven birthing hospitals and three-quarters of PNC practices do not initiate MOUD. Perhaps most notably, around two-thirds of responding birthing hospitals and PNC practices reported not offering any naloxone to patients.

While most SUD programs offering MOUD reported no restrictions related to pregnancy status, our data reveal several potential barriers to treatment engagement. About a quarter of SUD programs reported requiring obstetric evaluation prior to MOUD initiation, which may be an obstacle to the OUD stabilization needed to facilitate engagement in prenatal care. Additionally, few programs offer tailored services that can increase the relevance and accessibility of treatment for pregnant patients or for parents of young children. While most residential programs and recovery residences did report accepting patients during pregnancy, far fewer allow children onsite. (Of note, the higher rate of permitting children among recovery residences in our sample [50%] may be inflated by the low response rate among recovery residences and overrepresentation of programs specifically catering to this population.) Even among outpatient SUD providers, only about half permit children to accompany parents to treatment. These limits likely lead to a significant loss of treatment options after delivery for many patients given competing childcare responsibilities [13, 29].

Withdrawal management services for the perinatal population were also a notable gap within the Baltimore landscape. With regards to OUD, several of the dedicated withdrawal management/stabilization programs surveyed indicated denying buprenorphine management to pregnant patients when it was otherwise available to the general population. Alcohol/benzodiazepine withdrawal management, a critical service to facilitate safe cessation of these substances, was also unavailable in many locations, including SUD programs overall and particularly among PNC practices. This lack of availability may in part be due to the fact that these services, particularly for cases of severe withdrawal, often merit inpatient-level monitoring; however, even two of seven birthing hospitals and two of five alcohol/benzodiazepine withdrawal management programs reported denying this service during pregnancy.

Among the most commonly endorsed needs by respondents were education/specialist consultation on SUD treatment in pregnancy within obstetric settings and on pregnancy-related medical concerns within SUD programs. These endorsements mirror MMRC recommendations to increase PNC and SUD provider comfort caring for patients with perinatal OUD and to reduce silos between these disciplines [5, 31, 32]. Despite the limited availability of naloxone for overdose reversal among birthing hospitals and PNC practices, very few endorsed that they would benefit from the ability to distribute naloxone directly to patients. Lack of interest in providing this service may reflect an unawareness of the prevalence of overdose during the perinatal period as well as the potential benefit of directly distributed naloxone, compared with prescribed naloxone, in accessibility and reduction of overdose deaths [38–40].

Overall, our results highlight strengths and weaknesses within the landscape of perinatal SUD services in Baltimore and potential avenues for quality improvement. Within Baltimore, results from this project have served as a critical guide to our team’s ongoing capacity building efforts citywide. In particular, dissemination of results to multiple local professional organizations/networks and mortality review committees has offered the opportunity for education about maternal overdose mortality as well as potential ways to strengthen care for this population. Additionally, results have enabled the project team to identify and conduct targeted outreach and technical assistance to organizations that were either already providing care to this population or had the potential to fulfill an unmet need; 88 clinicians across 10 SUD treatment and/or prenatal care organizations have been engaged thus far. Depending on specific organization needs, outreach offerings have included provider education on standards of SUD care in pregnancy, promotion of population-specific community resources (e.g., case management, legal support, doula care), supported linkage to obstetric resources, and longitudinal consultation on development of population-specific programming. While this landscape assessment is specific to Baltimore City, the patterns identified may be relevant more broadly. This project may serve as a model for other jurisdictions interested in better understanding their local landscape and strengthening services for patients using opioids during pregnancy and the postpartum period.

Data from our project should be interpreted in light of both its strengths and limitations. Our assessment was unique in its comprehensiveness–both due to the broad multidisciplinary sample of organizations included as well as the range of topics addressed. Additionally, our systematic contact confirmation and outreach efforts yielded a strong response rate of 61%. However, not all organizations providing services to pregnant patients with OUD in Baltimore City are represented in this data. Some organizational categories, such as certified-recovery residences, yielded lower response rates than others, and so data about these programs may not be as broadly applicable. Additionally, some SUD-related organizations or providers, particularly those providing more informal care (such as non-certified recovery residences or individual buprenorphine prescribers outside of larger SUD-focused practices), may not have been captured by our outreach lists. For a small number of organizations on the initial outreach lists, our team was unable to identify individual respondents, leading to their exclusion. Additionally, those organizations with greater focus on this specialized population likely had a higher response rate, which may artificially increase the reported rate of service availability. Importantly, reported service availability may not accurately reflect true access to services, quality of care provided, and/or the patient experience receiving care. All of these factors may result in lower actual accessibility of services than is represented in our data.

## Conclusions

This project provides an extensive picture of health services available for perinatal OUD across birthing hospitals, prenatal care practices, and SUD treatment programs in Baltimore, Maryland. Results have illuminated strengths as well as gaps in the landscape of care available and have served to guide ongoing citywide system improvement efforts by our project team, including targeted outreach and technical assistance to organizations providing SUD treatment and/or prenatal care to patients with perinatal SUD. As the first known citywide, multidisciplinary service assessment to be published in the literature, this project offers a model for other jurisdictions hoping to strengthen perinatal health services for patients with OUD and reduce maternal mortality.

## Electronic supplementary material

Below is the link to the electronic supplementary material.

Supplementary Material 1

## Data Availability

The datasets used and/or analyzed during the current study are available from the corresponding author on reasonable request.

## Abbreviations

ASAM: American Society of Addiction Medicine
MMRC: Maternal mortality review committee
MOUD: Medication for opioid use disorder
OBOT: Office-based buprenorphine treatment
OTP: Opioid treatment program
OUD: Opioid use disorder
PNC: Prenatal care
SBIRT: Screening, brief intervention and referral to treatment
SSP: Syringe services program
SUD: Substance use disorder
