# Supplementary material for: Services for perinatal patients with opioid use disorder: a comprehensive Baltimore City-wide 2023 assessment
Source: Addict Sci Clin Pract. 2024 Oct 15;19:73. doi: 10.1186/s13722-024-00507-0 (PMC11476604; doi:10.1186/s13722-024-00507-0)
Supplement: Supplementary file 1 — Supplementary Material 1 [file 13722_2024_507_MOESM1_ESM.pdf]

Supplement *(skip patterns not represented)*

Baltimore City Perinatal SUD Treatment Landscape Assessment

About This Survey

Overdose is the leading cause of death for pregnant and postpartum people in Baltimore City. To improve care for this population, we are conducting a survey of substance use disorder (SUD) treatment programs, prenatal care clinics, and obstetrics units asking about services offered, referrals commonly made, and barriers to effective care.

This survey asks about services at your organization, not your personal health information. Your participation is voluntary and should take less than 15 minutes to complete. We will ask for your name and work contact information for possible follow-up, though you are not required to provide this information. Your personal information will not be shared. Data will be analyzed to describe the landscape of resources in Baltimore City to address perinatal opioid use disorder (OUD) and identify ways to strengthen the system of care. Local dissemination of results may include information about your organization to support systems improvement. Any dissemination of results beyond the local setting will not include organization names. Risks of participation include possible loss of confidentiality; there are not likely to be direct benefits to you from participation.

The survey is being conducted by the Opioid Overdose Prevention division and the Bureau of Maternal and Child Health at Baltimore City Health Department, in collaboration with Johns Hopkins Division of Addiction Medicine, as part of the B'more for Healthy Babies initiative. The project has been reviewed by the Johns Hopkins IRB and determined to be a quality improvement initiative. If you have any questions, contact Jessica Ratner at jratner1@jh.edu.

What is/are the primary mission(s) of your program/clinic/unit? Select all that apply. If none of these options are applicable, please stop survey and contact us.

- ☐ Services for individuals with substance use disorder
- ☐ Outpatient prenatal care
- ☐ Hospital-based obstetrics care

RESPONDENT INFORMATION

Program/Clinic/Unit Name

____

Respondent Name

____

Respondent Title/Role

____

SERVICES OFFERED

What ASAM level(s) of substance use disorder (SUD) treatment does your program provide? Select all that apply.	☐ Level 1 (Outpatient)<br>☐ Level 2.1 (Intensive outpatient)<br>☐ Level 2.5 (Partial hospitalization)<br>☐ Level 3.1 (Clinically managed low-intensity residential services)<br>☐ Level 3.3 (Clinically managed population-specific high-intensity residential services)<br>☐ Level 3.5 (Clinically managed high-intensity residential services)<br>☐ Level 3.7 (Medically monitored intensive inpatient services)<br>☐ Level 4 (Medically managed intensive inpatient services)<br>☐ Other/Not applicable (please describe) ______<br>☐ Unsure/Don't know
Does your program assess for pregnancy at intake for SUD-related services?	◯ Yes<br>◯ No<br>◯ Unsure/Don't Know
How does your program assess for pregnancy?	☐ Self-report<br>☐ Urine test<br>☐ Other (please describe) ______<br>☐ Unsure/Don't know
How often does your program have at least one pregnant individual enrolled for SUD treatment services?	◯ Always<br>◯ Frequently<br>◯ Sometimes<br>◯ Rarely<br>◯ Never<br>◯ Unsure/Don't know
In an average month, what percentage of your program's census consists of pregnant individuals? Please provide your best estimate.	0 50% 100%<br>*(Place a mark on the scale above)*
Does your clinic/unit assess for opioid use disorder (OUD) at intake for prenatal or obstetric services?	◯ Yes<br>◯ No<br>◯ Unsure/Don't Know
How does your clinic/unit assess for OUD? Select all that apply.	☐ Informal verbal/written screening<br>☐ Validated written/verbal screening tool (please specify) ______<br>☐ Urine toxicology testing<br>☐ Other (please describe) ______<br>☐ Unsure/Don't know
How often does your clinic/unit have at least one individual with OUD receiving care?	◯ Always<br>◯ Frequently<br>◯ Sometimes<br>◯ Rarely<br>◯ Never<br>◯ Unsure/Don't know
In an average month, what percentage of your clinic/unit's patient census consists of individuals with OUD? Please provide your best estimate.	0 50% 100%<br>*(Place a mark on the scale above)*

Please select answers below to describe the availability of the listed SUD-related treatments/services in your program, clinic or unit.

	Available to all individuals (regardless of pregnancy status)	Available to pregnant individuals only	Available to non-pregnant individuals only	Not available	Unsure/Don't know
Buprenorphine initiation	○	○	○	○	○
Buprenorphine maintenance	○	○	○	○	○
Methadone initiation	○	○	○	○	○
Methadone maintenance	○	○	○	○	○
Naltrexone initiation	○	○	○	○	○
Naltrexone maintenance	○	○	○	○	○
Withdrawal management/detox from opioids	○	○	○	○	○
Withdrawal management/detox from alcohol/benzodiazepines	○	○	○	○	○
Brief intervention/counseling (sometimes referred to as "SBIRT")	○	○	○	○	○
Peer recovery support	○	○	○	○	○
Non-peer treatment linkage services	○	○	○	○	○
Individual substance use counseling	○	○	○	○	○
Group substance use counseling	○	○	○	○	○
Naloxone - direct distribution of kits	○	○	○	○	○
Naloxone – providing prescriptions	○	○	○	○	○
Harm reduction services (ex: providing clean supplies)	○	○	○	○	○
Housing during treatment (residential level of care or supportive housing)	○	○	○	○	○
Other:______	○	○	○	○	○
Other:______	○	○	○	○	○

Which of the following substance use disorder (SUD)-related treatments/services does your clinic/unit offer? We recognize that some of the listed options may not be applicable to your practice setting. Select all that apply.	☐ Buprenorphine initiation<br>☐ Buprenorphine maintenance<br>☐ Methadone initiation<br>☐ Methadone maintenance<br>☐ Naltrexone initiation<br>☐ Naltrexone maintenance<br>☐ Withdrawal management/detox from opioids<br>☐ Withdrawal management/detox from alcohol/benzodiazepines<br>☐ Brief intervention/counseling (sometimes referred to as "SBIRT")<br>☐ Peer recovery support<br>☐ Non-peer treatment linkage services<br>☐ Individual substance use counseling<br>☐ Group substance use counseling<br>☐ Naloxone - direct distribution of kits<br>☐ Naloxone - providing prescriptions<br>☐ Harm reduction services (for example, providing clean supplies)<br>☐ Other (please describe below)<br>☐ None of the above
Please describe "other" service provided:	______________________________
What evaluation do you require prior to initiation of medications for OUD for a pregnant individual? Select all that apply.	☐ None<br>☐ Obstetric ultrasound<br>☐ Outpatient evaluation by obstetric provider<br>☐ Inpatient hospitalization<br>☐ Other (please describe) ______<br>☐ Unsure/Don't know<br>☐ Not applicable (we do not initiate medications for OUD for pregnant individuals)
What steps does your program take if an individual already enrolled in your program for SUD-related services becomes pregnant? Please be as specific as possible. If not applicable, write "N/A."	______________________________________
Please provide additional comments on availability of your program's services during pregnancy:	_____________________________________
Does your program offer any services specifically focused on pregnant or postpartum individuals? ("Postpartum" means within 1 year of birth, termination, or pregnancy loss.)	◯ Yes ◯ No ◯ Unsure/Don't know
If "yes," please describe:	_____________________________________
Does your program offer any services specifically focused on individuals with young children (under age 5)?	◯ Yes ◯ No ◯ Unsure/Don't know
If "yes," please describe:	_____________________________________

Are children permitted to be onsite with their legal guardian(s) during treatment? ◯ Yes ◯ No ◯ Unsure/Don't know

If "yes," please describe: __________________________________

REFERRALS AND PARTNERS

If your program receives referrals of pregnant and/or postpartum individuals with SUD, from which organizations, programs, or clinics do those referrals commonly come? Please be as specific as possible. If not applicable, write "N/A." __________________________________

From which organizations, programs, or clinics are individuals commonly referred to your clinic for prenatal care, if known? Please be as specific as possible. __________________________________

If medications for OUD (that is, methadone, buprenorphine and/or naltrexone) are not available to pregnant individuals at your program/clinic do you refer them elsewhere for these medications? If so, to which programs? If not applicable, write "N/A." __________________________________

To which other programs does your program refer if a pregnant individual needs a higher level of SUD care (if applicable)? For example, for inpatient stabilization or detoxification, residential level services, or treatment with housing. If not applicable, write "N/A." __________________________________

To which providers/practices does your program refer pregnant individuals for prenatal care? How does your program make this referral? Please be as specific as possible. If not applicable, write "N/A." __________________________________

To which clinics/practices does your clinic/unit refer individuals with SUD for ongoing healthcare after pregnancy? How does your clinic/unit make this referral? Please be as specific as possible. __________________________________

With what other organizations, programs, health clinics, etc. does your program/clinic/unit partner to provide care for pregnant or postpartum individuals with SUD? Please be as specific as possible. __________________________________

BARRIERS AND NEEDS

List the top barriers (up to 3) to caring effectively for pregnant/postpartum individuals with SUD. For example, there may be barriers related to workforce, finance/insurance, knowledge gaps, stigma/discrimination, access issues, etc. Please be as specific as possible.

____

What information or resources would enable your program/clinic/unit to better serve this population? Select all that apply.

- ☐ Education on meds for OUD in pregnancy
- ☐ Education on other SUD treatment in pregnancy
- ☐ Education on pregnancy-related medical concerns
- ☐ Specialist consultation for SUD treatment in pregnancy
- ☐ Specialist consultation for pregnancy-related medical concerns
- ☐ Support with workflow/policy implementation (please describe below)
- ☐ Onsite sexual health services provided by external partner
- ☐ Onsite SUD treatment services provided by external partner
- ☐ Increased availability of specific resources for patients [e.g., social services, healthcare resources] (please describe below)
- ☐ Ability to distribute Naloxone to individuals directly
- ☐ Other (please describe below)
- ☐ Unsure/don't know
- ☐ None of the above

Please describe workflow/policy implementation support needed:

____

Please describe "specific resources" needed for patients:

____

Please describe "Other":

____

ADDITIONAL COMMENTS

Please write any additional comments here:

____

FOLLOW-UP

If needed, may we contact you with follow-up questions?

◯ Yes ◯ No

What is the best contact information for reaching you?

____
